# T cell immunity to glatiramer acetate ameliorates cognitive deficits induced by chronic cerebral hypoperfusion by modulating the microenvironment

**DOI:** 10.1038/srep14308

**Published:** 2015-09-22

**Authors:** Li Chen, Yang Yao, Changjuan Wei, Yanan Sun, Xiaofeng Ma, Rongxin Zhang, Xiaolin Xu, Junwei Hao

**Affiliations:** 1 Department of Neurology and Tianjin Neurological Institute, Tianjin Medical University General Hospital, Tianjin 300052, China; 2 Center for Basic Medical Science Research, Tianjin Medical University, Tianjin 300070, China; 3 Department of Neurology, Tianjin Huanhu Hospital, Tianjin 300060, China

## Abstract

Vascular dementia (VaD) is a progressive and highly prevalent disorder. However, in a very large majority of cases, a milieu of cellular and molecular events common for multiple neurodegenerative diseases is involved. Our work focused on whether the immunomodulating effect of glatiramer acetate (GA) could restore normalcy to the microenvironment and ameliorate cognitive decline induced by chronic cerebral hypoperfusion. We assessed cognitive function by rats’ performance in a Morris water maze (MWM), electrophysiological recordings and by pathologic changes. The results suggest that GA reduced cognitive deficits by reestablishing an optimal microenvironment such as increasing expression of the brain-derived neurotrophic factor (BDNF) and modulating the Th1/Th2 cytokine balance in the hippocampus. When microenvironmental homeostasis is restored, cholinergic activity becomes involved in ameliorating cellular damage. Since vaccination with GA can boost “protective autoimmunity” in this way, a similar strategy may have therapeutic potential for alleviating VaD disease.

Vascular dementia (VaD), which is second only to Alzheimer’s disease (AD) as a source of cognitive decline, has gained a notable increase in universal attention^1^. VaD is characterized by a progressive cognitive and behavioral deterioration induced by focal, multifocal or diffuse vascular and/or ischemic lesions involving various brain areas and neuronal networks^2^. Central to the disease-initiating mechanism is damage mainly to the cerebral blood vessels. The result is not only delivery to the brain of insufficient oxygen and nutrients but also disruption of microenvironmental homeostasis at the molecular level. Some manifestations are an increase in the reactive gliosis^3^, pro-inflammatory cytokines^4^ as well as a decrease in neurotrophic factors^5^. In fact, defects of the cholinergic nervous system have been proposed as the most vital cause of human dementia, leading to formulation of the cholinergic hypotheses of senile dementia^6^,^7^,^8^ and at least partly responsible for the related cognitive decline. Bilateral permanent occlusion of the common carotid arteries (2VO) in rats is a well-characterized model that exhibits many features resembling those found in human VaD^9^. In this context, we theorized that a decrease in “microenvironment homeostatic reserve” may lead to cellular malfunction, even apoptosis, in which cholinergic neuron dysfunction is involved.

Traditionally, immune activity in the central nervous system (CNS) had been considered detrimental; therefore, the CNS was considered an “immunologically privileged site”^10^. However, numerous studies have demonstrated that cells of the immune system may play an essential role in protecting the injured CNS or offsetting neurodegenerative effects^11^. CNS-specific CD4+ T cells, which circulate in the cerebrospinal fluid, meninges and choroid plexus and are available for immunosurveillance can improve spatial learning and memory performance^12^,^13^. That phenomenon was called “protective autoimmunity” by Schwartz and collaborators. In accord, the T cell response specific for brain proteins was found capable of increasing the expression of neurotrophic factors^14^, enhancing memory and synapse plasticity^15^,^16^, increasing the buffering capacity of glutamate^17^ and facilitating the recruitment of blood monocytes that locally displayed an anti-inflammatory role in restoring homeostasis in CNS maintenance^18^. Therefore, a desirable goal is the establishment of immune neuroprotection free from autoimmune disease. To this end, the efficacy of the neuroprotective autoimmune response may depend upon well-controlled timing, intensity as well as the nature of the disease^14^,^19^,^20^.

Glatiramer acetate (GA), an approved drug for the treatment of multiple sclerosis, consists of acetate salts of synthetic polypeptides containing L-alanine, L-glutamate, L-lysine and L-tyrosine. The beneficial characteristics of GA were previously demonstrated: (1) like myelin basic protein (MBP), GA can weakly cross-react with CNS-resident autoantigens and simulate the protective and reparative effects of autoreactive T cells^14^,^19^,^21^. (2) GA is a weak agonist to self-antigens, but it basically induces a systemic immune response without the risk of autoimmune disease, active immunization with this drug is safe^11^. (3) GA-induced T cells have been shown to home to brain tissue^14^. Strikingly, GA promoted an immune-mediated neuroprotective response in several models of acute CNS injury and neurodegenerative disease such as AD, Parkinson’s disease (PD), amyotrophic lateral sclerosis (ALS) and depression, etc.^11^. However, the effect of GA on VaD is still unclear.

Since chronic cerebral hypoperfusion can injure the local immune microenvironment and damage the CNS at sites of cholinergic activity associated with learning and memory, we evaluated here whether dementia induced by chronic cerebral hypoperfusion could be ameliorated by GA immunization, thereby restoring the neurogenic microenvironment and regulating cholinergic dysfunction during 2VO of rat models.

## Results

### Effect of GA in Morris water maze (MWM) behavioral tests

**Figure 1:**
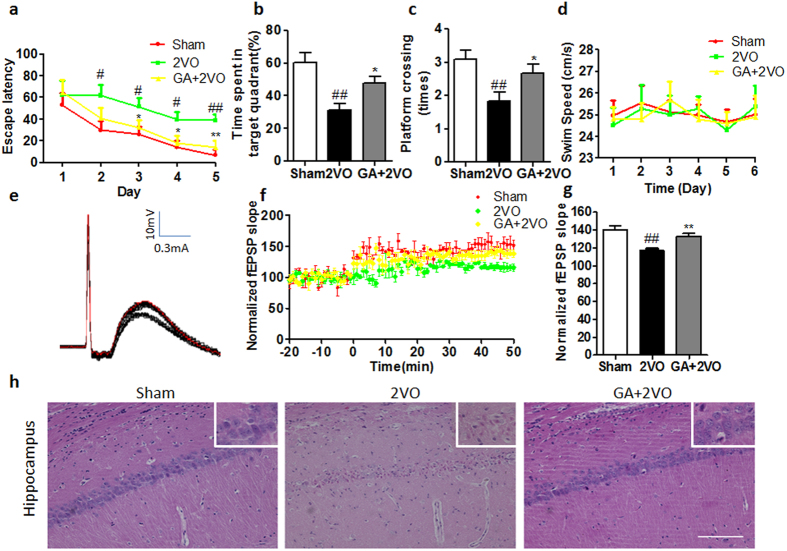
Effects of GA on cognitive deficits of rats induced by chronic cerebral hypoperfusion: behavioral, LTP and cellular level changes. (**a**) Average escape latency to find the target platform in MWM test. The 2VO group spent more time finding the hidden platform on day 2 (*P* < 0.05), day 3 (*P* < 0.05), day 4 (*P* < 0.05) and day 5 (*P* < 0.01) in the training phase than the sham-operated group, indicating the cerebral hypoperfusion significantly impaired spatial learning in rats. GA treatment significantly decreased the latent period to locating the escape platform compared with the PBS-treated 2VO group on days 3 (*P* < 0.05), day 4 (*P* < 0.05) and day 5 (*P* < 0.01) of training. n = 12 (**b,c**) The percentage of time spent in the target quadrant within 60 sec and platform crossing times in the probe trial. The 2VO group spent less time in the target quadrant and less numbers of platform crossing than the sham group (*P* < 0.01, *P* < 0.01 respectively). GA-treated rats spent more time in the target quadrant and more numbers of platform crossing than did the PBS-treated 2VO rats (*P* < 0.05, *P* < 0.05 respectively). (**d**) There was not significantly difference among the three groups in terms of the mean swimming velocity in MWM (*P* > 0.05). (**e**) Representative electrophysiological profile. (**f,g**) LTP levels and time course changes in fEPSP slope in three groups respectively. In 2VO group, the normalized slope of the fEPSP was significantly reduced compared with that in sham group (*P* < 0.01). However, GA could attenuate this change compared with that in 2VO group (*P* < 0.05). n = 8 (**g**) Representative photographs of tissue sections stained with H&E in the hippocampal CA1 area (magnification: 200×). Section from a sham-operated group shows intact neurons and well-preserved cell density. Section from the 2VO group shows neuronal loss with ischemic changes (neuronal cell loss, nuclei shrinkage and dark staining of neurons). Section from the GA group shows more or less intact neurons with intact chromatin. Scale bar = 50 μm, n = 4. The datas of average escape latency were analyzed with two-way repeated ANOVA and other datas were analyzed by one-way ANOVA; shown are the means ± SEM. (*P < 0.05 and **P < 0.01 vs 2VO group, #*P* < 0.05 and ##*P* < 0.01 vs sham group).

MWM test is one of validate behavioral method to evaluate spatial learning memory. After sham surgery or the induction of injury to the CNS by neurosurgery (2VO) followed by 3 weeks GA or PBS injection, the spatial learning memory of 2VO rats with and without GA-treatment was compared by using MWM testing. Two-way ANOVA for repeated measures indicated that the 2VO groups achieved longer latency times than the sham-operated group on day 2 (*P* < 0.05), day 3 (*P* < 0.05), day 4 (*P* < 0.05) and day 5 (*P* < 0.01). Notably, 2VO rats treated with GA had significantly shorter latency swim times than did 2VO rats treated with PBS on day 3 (*P* < 0.05), day 4 (*P* < 0.05) and day 5 (*P* < 0.01) of training(see Fig. 1a). Moreover, One-way ANOVA suggested that the 2VO group spent less time in the target quadrant than the sham group (*P* < 0.01), and the percentage of time spent in the target quadrant was significantly higher for GA-treated rats than for 2VO-PBS controls (*P* < 0.05) (see Fig. 1b ). In addition, GA treatment increased the numbers of times that rats crossed the platform areas than 2VO groups (*P* < 0.05) (see Fig. 1c). However, there is no difference in swimming speed among the sham group, 2VO group and GA treated group (*P* > 0.05) (see Fig. 1d). Taken together, these results demonstrated that GA treatment could ameliorate the 2VO behavioral impairment due to improving memory rather than differences in motor ability.

### Effect of GA on electrophysiological assessment with recording of long-term potentiation (LTP) in hippocampus

After the MWM test, the LTP at CA3-CA1 synapses in hippocampus was recorded, which was considered as one of the functional indexes of cognition. LTP was evaluated as mean percentage change in the amplitude of the population spike following high frequency stimulation (HFS) compared to its baseline. Fig. 1e showed a representative electrophysiological profile. As shown in Fig. 1f,g, the field excitatory postsynaptic potential (fEPSP) slope was 139.33 ± 1.76%, 114.33 ± 1.67%, 133.33 ± 3.28% in sham group, 2VO group and GA group respectively. In 2VO group, the normalized slope of the fEPSP was significantly reduced compared with that in sham group while GA treatment could attenuate this change compared with that in 2VO group(*P* < 0.01 2VO group vs sham group; *P* < 0.01 GA group vs 2VO group). This indicated the T cell immunity to GA could attenuate the synaptic dysfunction.

### Effects of GA on hematoxylin and eosin (H&E) stain analysis of the hippocampal CA1 area

H&E strain is one of important measure to evaluate neuronal loss morphology in rats. We focused on the hippocampus CA1 area, as this limbic area involved in learning and memory is particularly sensitive to hypoxia-ischemia conditions.The Sham group showed intact neurons in the CA1 area of the hippocampus. In contrast, extensive neuronal changes in this area were visible in 2VO rats; i.e., thickening decrease, neuronal cell loss, nuclei shrinkage and dark staining of neurons. However, GA markedly reduced these pathological changes and attenuated cell loss induced by chronic cerebral hypoperfusion of treated rats(Fig. 1h).

### Effect of GA on brain-derived neurotrophic factor (BDNF)

**Figure 2:**
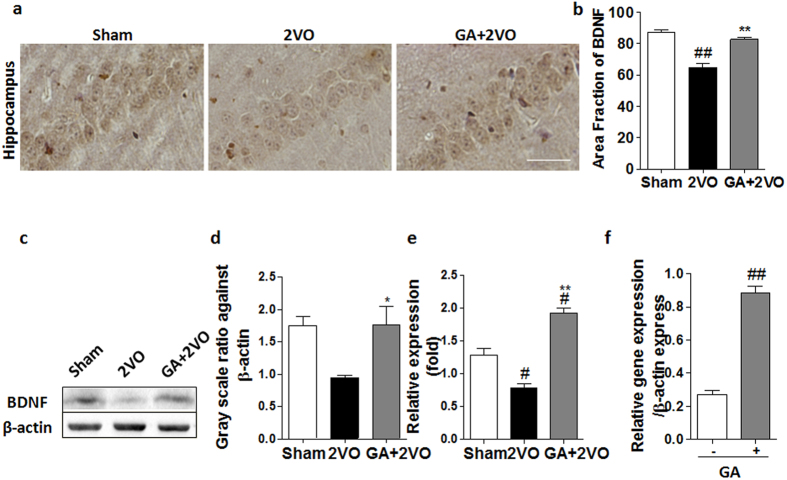
GA treatment increased the level of BDNF in hippocampus of 2VO rats. (**a,b**) As in representative immunohistochemical micrographs with statistical analysis, levels of hippocampal BDNF increased after GA treatment (magnification: 200×). Scale bar = 50 μm. n = 4 (**c,d**) Western blots of BDNF expression in the hippocampus and histograms show quantification of the GA effect on BDNF in the hippocampus. n = 5 (**e**) GA up-regulated the mRNA expression of BDNF in the 2VO model. n = 5 (**f**) Ex-vivo, secretion of BDNF by GA-reactive T cells increased significantly after stimulation of T cells with GA (50 ug/ml) compared to unstimulated T cells. The data of ex-vivo experiments of BDNF was tested for normality and evaluated by Student´s t-test (T) and other datas were subjected to ANOVA.The means ± SEM are shown (*P < 0.05 and **P < 0.01 as compared to 2VO group).

BDNF is a member of neurotrophic family of nerve growth factors, a key protein in promoting memory, growth and survival of neurons^22^. As shown in Fig. 2a,b, representative immunohistochemical micrographs and statistical analysis determined that GA treatment increased the level of BDNF in the hippocampus. GA-treated rats had increases of BDNF-positive neurons compared with that in 2VO rats (*P* < 0.01), but nearly approximated amounts in sham-operated rats. In Fig. 2c,d, Western blots denoted increases of BDNF expression in the hippocampuses of GA-treated rats compared with that in 2VO rats (*P* < 0.05), but approximated that in sham rats. Fig. 2e presented further analyses of mRNA expression in the hippocampus as carried out with quantitative real-time polymerase chain reaction (qPCR). The GA-treated group had increased BDNF mRNA levels compared with that in the 2VO group (*P* < 0.01). Taken together, we demonstrated GA could increase the BDNF in the hippocampus from the mRNA and protein expression level respectively. Additionally, It has been reported that the GA-reactive T cells could home to pathologic sites in the CNS^14^,^23^ and produce BDNF^14^,^23^,^24^. We therefore carried out an ex-vivo experiment to determine whether T cells on encountering GA can upregulate BDNF transcripts. The qPCR analysis of BDNF secreted by T cells compared to unstimulated T cells (Fig. 2f) revealed that secretion of BDNF by GA-reactive T cells was significantly increased after stimulation of the T cells with GA (*P* < 0.01).

### Effect of GA on the expression of Ionized calcium bindingadaptor molecule-1 (Iba-1) and glial fibrillary acidic protein (GFAP)

**Figure 3:**
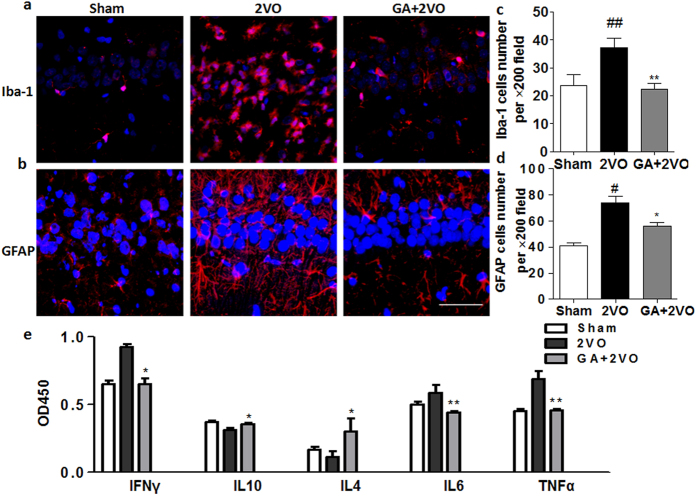
GA treatment attenuated inflammation induced by chronic cerebral hypoperfusion via modulation of the Th1/Th2 balance. (**a,c**) The expression of Iba-1 in the hippocampus, immunostaining for Iba-1 (red). (**b,d**) The expression of GFAP in the hippocampus of rats, immunostaining for GFAP (red). The expression of Iba-1 and GFAP was observed in all three treatment groups. The 2VO group showed increased gliosis compared to the sham group, whereas the GA treatment group showed a decreasing expression of Iba-1 and GFAP compared to the 2VO group (*P* < 0.01 and *P* < 0.05, respectively) (magnification 200×). Scale bar = 50 μm. (**e**) ELISA measurement of cytokine profiles. The concentrations of IFN-γ, IL-4, IL-10, IL-6, TNF-α were determined in supernatants of hippocampal tissues by ELISA. Amounts of IFN-γ, IL-6 and TNF-α were inhibited, but levels of IL-4 and IL-10 in the GA-treatment group exceeded those in the 2VO group, although not to a significant extent compared with the sham group. n = 4. Data were analyzed by ANOVA. Means are shown ± SEM (*P < 0.05 and **P < 0.01 as compared to 2VO group).

Ample evidences have shown that chronic cerebral hypoperfusion may induce glial activation in the brain^3^. In agreement with these studies, our findings showed that Iba-1- and GFAP-positive cells were rare in the hippocampus of rats in the sham-operated group (Fig. 3a–d). However, activated microglia cells and astrocytes were markedly increased in the hippocampus of the 2VO group. Administration of GA reduced the number of Iba-1- and GFAP-positive cells in the hippocampus of 2VO rats below that in the 2VO group (*P* < 0.01 and *P* < 0.05, respectively), which indicated that GA inhibited astrocytosis and microgliosis.

### Effects of GA treatment on cytokine profiles

The effects of GA treatment on the cytokine profiles produced in supernatants of hippocampus tissue were analyzed semi-quantitatively using multi-cytokine ELISA kits. It has been demonstrated that the whole lymphocyte populations isolated from brains, lymph nodes and spleens reactive to GA can increase interleukin 4 (IL-4), interleukin 5 (IL-5), interleukin 10 (IL-10) and tumor growth factor β (TGF-β)^25^,^26^. So we wanted to investigate the changes of whole immune microenvironment in hippocampus tissue. As shown in Fig. 3e, the production of interferon-γ (IFN- γ), interleukin 6 (IL-6) and tumor necrosis factor α (TNF-α) was significantly reduced in the GA-treated groups compared to those levels in the 2VO group (*P* < 0.05, *P* < 0.01, *P* < 0.01 respectively), whereas amounts of IL-4 and IL-10 were upregulated (*P* < 0.05 and *P* < 0.05, respectively).

### Effect of GA on central cholinergic activity

**Figure 4:**
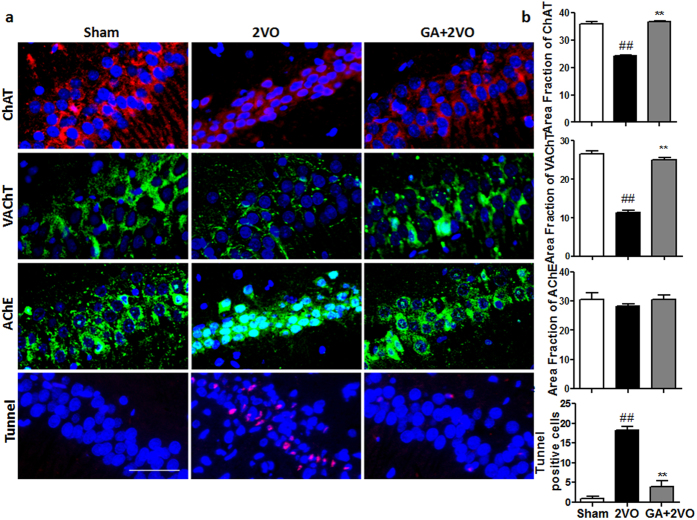
GA treatment increased the production of cholinergic activity in the CA1 area of the hippocampus in 2VO rats (magnification: 200×). (**a**) Representative images of cholinergic activity and neurocyte apoptosis in the hippocampal CA1 area as seen by fluorescence microscopy: immunostaining for ChAT (red), immunostaining for VAChT (green), immunostaining for AChE (green) and immunostaining for Tunnel (red). (**b**) Quantification of GA’s effect on ChAT, VAChT, AChE and Tunnel-positive cells. As visible in the GA group, the decrease of ChAT and VAChT activity induced by cerebral hypoperfusion are significantly alleviated compared to that in the 2VO group (*P* < 0.01); however, the effect on AChE activity was not significant (*P* > 0.05). At the same time, administration of GA prevented the apoptosis of neurons. Scale bar = 50 μm. n = 4. Data were analyzed by ANOVA, shown are means ± SEM (*P < 0.05 and **P < 0.01 as compared to the 2VO group, #*P* < 0.05 and ##*P* < 0.01 as compared to the sham group).

The central cholinergic systems play an important role in cognitive function. As indirectly demonstrated, 2VO reduced the level of acetylcholine (ACh) in rat brains^27^. As shown in Fig. 4, we found that 2VO induction caused a significant decrease of choline acetyltransferase (ChAT) and vesicular acetylcholine transporter (VAChT) activity in the hippocampus compared to that in the sham-operated group (*P* < 0.01 and *P* < 0.01, respectively), indicating the impairment of cholinergic function by chronic cerebral hypoperfusion. However, GA significantly hindered the decrease of ChAT and VAChT activity induced by cerebral hypoperfusion compared with those levels in the 2VO group (*P* < 0.01 and *P* < 0.01, respectively), whereas the content of the acetylcholinesterase (AChE) had no observable change. These results indicated that GA can increase cholinergic activity. In addition, we detected Tunnel-positive material localized in the neurons’ nuclei. Compared with 2VO group, GA-treated rats showed a significant reduction of Tunnel-positive neurons in the hippocampal CA1 area (*P* < 0.01). Overall these results suggested that GA treatment might decrease apoptosis.

## Discussion

This study clearly indicated that chronic cerebral hypoperfusion, a dementia-inducing phenomenon, causes cognitive deficits characterized by marked behavioral, electrophysiological and cellular changes. Here, these injurious effects were ameliorated by GA treatment with a parallel restoration of the microenvironment, including an increased expression of BDNF, modulation of the Th1/Th2 balance and upregulation of the cholinergic system.

To investigate cognitive function in an animal model that simulates human VaD in humans, we used the MWM paradigm, which is a widely accepted behavioral method for assessing learning and memory in rodents^28^. In our study, rats were subjected to bilateral permanent occlusion of the common carotid arteries (i.e., 2VO) followed by chronic cerebral hypoperfusion, which caused learning and memory impairment. The results of subsequent MWM testing showed that the rats treated with GA achieved markedly better scores in learning and memory performance than their counterparts that did not receive GA.

Subsequently, LTP was done in hippocampus as LTP of electrophysiological recordings is considered as one of the functional indexes of synaptic plasticity, which is a widely accepted model for learning and memory at the cellular level^29^. LTP in the hippocampus is considered to be essential for cognition and the severity of dementia has been shown to correlate mainly with synapse dysfunction and loss more than with neuronal death^30^,^31^. A decrease LTP of the 2VO group was detected compared with that in sham group and the GA could alleviate LTP decreasing in the hippocampus compared with that of 2VO group. These data indicated that GA could attenuate local neural circuits dysfunction and maintain the synaptic plasticity.

Since 2VO can reduce blood flow in the brain to one-third of its normal value^9^, thereby causing selective neuronal injury in this vulnerable region, particularly in the hippocampus^32^, our H&E staining of hippocampus showed that the 2VO group given GA had a decrease in thickening of hippocampal tissue as well as fewer neurons lost, less shrinkage of nuclei, reduced cerebral edema and diminished darkening of neurons in CA1 field than in the sham-operated group, and that the administration of GA attenuated neuronal damage induced by the 2VO.

Emerging evidence has demonstrated the importance of well-functioning adaptive immunity in maintenance of the brain^33^. The Schwarzt group described GA as a “universal antigen” that weakly activates a wide spectrum of self-reactive T cells and boosts a well-controlled “protective autoimmunity”. According to the theory of protective autoimmunity, a distinction must be made between the self-recognizing, supportive immune components and autoimmune diseases that emerge when control is lost^34^. Moreover, it is interesting to note that the cognitive dysfunction common in the elderly or patients with acquired immune deficiency syndrome (AIDS), both of whom typically suffer a disproportionate reduction in immunity, could be interpreted as representing the relationship between immunity and cognition^35^.

One mechanism by which GA maintains CNS spatial learning/memory may be the upregulation of BDNF mRNA expression and protein content. BDNF, a member of neurotrophic family of nerve growth factors, is beneficial for normal cognitive ability, synaptic plasticity memory and survival of neurons^36^. A deficiency of BDNF previously observed in VaD patients was also present our current study. That is, the chronic cerebral hypoperfusion of rats introduced here reduced BDNF content in terms of both mRNA and protein expression levels. However, GA vaccinated into rats *in vivo* promoted the production of BDNF. Upon activation with GA ex-vivo, T cells also manifested greatly enhanced BDNF mRNA production. To some extent, this result indicates that GA is a source of BDNF induction in T cells. Independent studies have shown that GA treatment of not only T cells but also local glial cells can promote BDNF expression in both^37^,^38^,^39^. Furthermore, in line with this notion, was the previous findings that levels of BDNF expression were reduced in immune-deficient mice and in mice deficient in CNS-specific T cells yet were elevated in T cells specific to myelin basic protein (TMBP) in mice^40^.

Here, T cell immunity to GA effectively restored the brain’s immune microenvironment by the action of cytokines. The CNS-specific T cell maintians the protective benefits without the risk of autoimmune disease at least partly lies on its effective modulation of immune cell network with the capacity for self-resolution, when activated by danger signals^41^. We found that such immune activation was overwhelming in the 2VO group, including in gliocytes (such as astrocytes and microglia), and pro-inflammatory factors (such as TNF-α and IL-6) increased in the hippocampus with subsequent neuronal damage, even apoptosis. Excessively activated glial cells are major sources of pro-inflammatory factors (i.e. TNF-a, IL-6), and over-expression of either TNF-α or IL-6 play a disturbing role in learning and memory^42^,^43^. However, it should be noted that TNF-α, in addition to its neurotoxicity in certain conditions, may also display neuroprotective effects in selected signaling contexts^44^,^45^,^46^. Deregulation of cytokines (Th1 versus Th2) has been reported to be involved in the pathogenesis of many human diseases such as AD, PD, sleep disturbance, major depression, autoimmune diseases and other disorders^47^,^48^,^49^. Therefore, we determined the Th1/Th2 balance in VaD by measuring IFN-γ as the Th1 and IL-10 and IL-4 as the Th2 cytokines. Prior works had shown that GA-reactive T cells, generated in the periphery, readily crossed the blood/brain barrier and exerted therapeutic effects for damaged neural tissue^14^,^20^,^50^. Moreover, GA-specific T cells secreted IL-4, IL-5, IL-10 and TGF-β^51^,^52^,^53^, providing evidence for the induction of an anti-inflammatory Th2 phenotype to suppress innate immunity in affected brain tissue. In our work, VaD induced a decrease in IL-4 and IL-10 but an increase in IFN-γ in the hippocampus; however, GA treatment normalized the cytokine levels. Together, our results illustrated the possibility that GA directed local immune cells towards an anti-inflammatory state by shifting to the Th2 mode, a properly regulated immune suppression response.

The precise mechanisms involved in VaD remain unclear, but considerable evidence indicates that cholinergic deficits play a vital role, including the synthesis and degradation of ACh, ACh receptor deficits and cholinergic signal transduction defects, etc.^54^,^55^,^56^. Moreover, cholinergic therapies, mostly based on the “cholinergic hypothesis,” have shown promising effects with respect to cognitive improvement in VaD patients. With regard to cognition, ACh is a necessary modulator of acquisition, encoding, consolidation, reconsolidation, extinction, retrieval and expression^57^. Based on prior studies and the present results, we theorize that chronic cerebral hypoperfusion destroys the microenvironment and contributes to cellular damage involving a cholinergic deficit. Therefore, we focused here, on the cholinergic terminals and detected ChAT, VAChT and AChE activity. ChAT, the biosynthetic enzyme for ACh, is presently the most specific cholinergic marker for checking the functional state of cholinergic neurons in the CNS and peripheral nervous system^58^,^59^. AChE, the hydrolyzing enzyme for ACh, determines the duration of ACh action in the synaptic cleft^60^. So the dynamic concentration of ACh in cholinergic synapses in the VaD-afflicted brain is regulated by the expression and activation of ChAT and AChE^61^. VAChT is responsible for the transport of ACh from the cytoplasm into the synaptic vesicles, where it is stored until release. VAChT activity may affect generation of the readily releasable ACh pool^62^. We found that chronic cerebral hypoperfusion induced a significant decrease in ChAT and VAChT activity in ischemic brain tissues. In another words, GA enhanced the ACh biosynthetic and stored function. Although AChE activity did not change significantly, the number of neurons decreased. Cytotoxicity is known to enhance AChE activity^63^, but chronic cerebral hypoperfusion, itself, could lead to neuronal death and numerical reduction of the neurons. This result was the mixed consequence of the two foregoing causes. Therefore, GA treatment may upregulate ChAT and VAChT by reducing the apoptosis of neurons, which could compensate for the reduced ACh levels in brains of 2VO model mice and may have facilitated recovery of their ischemia-induced memory function.

## Conclusion

Our present findings indicate that GA, as a CNS-related peptide with the ability to activate weakly self-reactive T cells, ameliorated the cognitive deficits induced by chronic cerebral hypoperfusion and CNS cholinergic dysfunction. GA reestablished an optimal microenvironment with increased BDNF expression and modulation of the Th1/Th2 balance in the hippocampus stromal environment. In this context, GA is a likely candidate as an adjunct therapy for VaD.

## Experimental Procedures

### Experimental animals

Male Sprague-Dawley rats, 280–300 g, were purchased from the Vital River Corporation (Beijing, China). All animals were acclimated to the environment under temperature-controlled conditions and a 12 h light/dark cycle for 1 week with sufficient food and water. The study was carried out in accordance with the Declaration of Helsinki and with the Guide for Care and Use of Laboratory Animals as adopted and promulgated by the United National Institutes of Health. All experimental protocols were approved by the Animal Ethics Committee of the Tianjin Medical University.

### GA treatment

After 2 weeks of 2VO, animals were injected (s.c.) four times with a total of 100 μg GA (Teva Inc., Petah Tiqva, Israel) dissolved in 200 μL of PBS. During the first week, each rat received two injections separated by a 24 h interval and once per week for the following two weeks. The dose and treatment of GA were chosen based on published data^64^.

### Surgery and grouping

Briefly, to provide 2VO-operated animals, individual rats were anesthetized with chloral hydrate (350 mg/kg, i.p.); both carotid arteries were gently separated from the vagus nerve, and permanent artery occlusion was implemented with silk thread. Sham-operated rats received the same surgical procedure without the occlusion of double ligation. Two weeks later, the MWM was carried out to evaluate the cognitive deficit and motor ability for each 2VO rat (date not shown). For verification, rats in the 2VO group were randomly subdivided into a control 2VO Group (2VO rats treated with PBS) and a GA + 2VO Group (2VO rats treated with GA). A third group consisted of sham-operated rats treated with PBS.

### MWM test

Spatial learning memory was evaluated by rat’s performance in the MWM after five weeks of 2VO. The MWM test was conducted daily for 5 days. Each trial lasted until the rat being tested located the hidden escape platform within 2 min or less. If unsuccessful, the rat was guided by an experimenter to the hidden platform for 2 sec. Escape latency was recorded as an assessment of spatial memory. After the last learning trial, i,e., on day 6, a probe trial was conducted to evaluate spatial memory. The platform was removed from the water, and each rat was allowed to swim freely for 60 sec. The percentage of the time each animal remained in the target quadrant and platform crossing were calculated as an index of its spatial memory. Besides, the swimming velocity was recorded to assess the motor ability for 6 days.

### Electrophysiological recordings

After the MWM test, each rat was gently placed in the head stereotaxic apparatus (Narishige, Japan) after anesthetized with 30% urethane (0.4 ml/kg). To insert stimulating and recording electrodes (Advent Co., UK), the rat’s skull was drilled a round skull window (5 mm in diameter) by surgical tweezers. The tip of the recording electrode was inserted 3.5 mm posterior to bregma and 2.5 mm lateral to the midline. The stimulating electrode was positioned into 4.2 mm posterior to bregma and 3.5 mm to the midline. The electrodes were gently inserted into the hippocampus, with a depth at approximately 2.5 mm beneath the pia mater for the stimulation electrode and 2 mm for the recording electrode. The test single stimuli were delivered to the CA3 region that evoked a response of 50% of its maximum ranging from 0.1 mA to 1.0 mA. Subsequently, stable baseline was recorded every 30 s for 20 min. Finally, the fEPSP was recorded every 60 s for 50 min (Scope Software, Powlab, ADInstruments, Australia) after a HFS was applied (200-Hz trains, 10 pulses/train every 2 s, repeated 10 times). The fEPSP slope was used to measure synaptic efficacy by normalization to the baseline.

### Neuropathological analysis

After the LTP, rats from each group were immediately perfused with cold (4 °C) PBS for 2 min. The brains were removed rapidly and fixed in 4% paraformaldehyde overnight at 4°C, followed by embedding in paraffin. H&E staining is a routinely used method for evaluating brain tissue morphology in rats, so 8 μm thick coronal sections from these brains were stained with H&E (Solarbio Science & Technology, Beijing, China) to evaluate the degree of damage to brain tissues, and the morphology was judged by image analysis using a Nikon Coolscope (×10 and ×100 magnification; Nikon, Dusseldorf, Germany).

Immunohistochemistry was performed to assess the BDNF level. Paraffin-immobilized sections of the brain tissues were fixed in 3% hydrogen peroxide for 10 min at room temperature and then exposed to bovine serum albumin for 30 min. Sections were subsequently incubated with rabbit anti-rat BDNF antibody (1:1000; Merck Millipore Corporation, Darmstadt, Germany) overnight at 4 ˚C, then stained with rhodamine-conjugated AffiniPure goat anti-rabbit secondary antibody (1:200; Jackson ImmunoResearch, Pennsylvania, USA) for 1 h at 4 ˚C.

Immunofluorescent staining was used to evaluate the central cholinergic activity and content of Iba-1, GFAP in rats. Briefly, the brain sections were deparaffinized, rehydrated, boiled in a microwave oven for antigen retrieval (microwave method) and finally treated with 0.5% Triton X-100 for 1h at room temperature. For the detection of choline acetyltransferase (ChAT), we used goat anti-ChAT antibody (1:100, Merck Millipore Corporation, Darmstadt, Germany) and for VAChT, AChE, Iba-1 and GFAP, the rabbit anti-VAChT antibody (1:100, Abcam Biotechnology, Cambridge, UK), rabbit anti-AChE antibody (1:100, Abcam Biotechnology, Cambridge, UK), rabbit anti-Iba-1 antibody (1:200, Abcam Biotechnology, Cambridge, UK,) and rabbit anti-GFAP antibody (1:1000, Abcam Biotechnology, Cambridge, UK), respectively, all of which were applied overnight at 4°C . Sections were incubated with goat anti-rabbit (1:200; Jackson, PA, USA) and donkey anti-goat secondary antibodies (1:200, Invitrogen, Carlsbad, CA, USA), respectively, for 1h at 4 °C in the dark. Finally, images of sections were captured by fluorescence microscopy.

With Tunnel staining of tissues, we identified apoptotic cells. According to the manufacturer’s protocol (Roche Corporation, Basle, Switzerland), the sections were pre-treated with 4 °C acetone for 7 min at room temperature and then washed with PBS. Afterward, the sections were incubated in Tunnel reaction mixture for 1 h at 37°C followed by red staining to locate apoptotic nuclei.

### Western blot analysis

When the rats were sacrificed, the hippocampus was separated from the brian immediately and snap frozen in liquid nitrogen. Proteins were extracted from the hippocampus. Protein extracts were immunodetected with specific primary antibodies for the detection of BDNF (1:1000; Merck Millipore Corporation, Darmstadt, Germany) and for the detection of β-actin (1:5000, Sigma-Aldrich, Milwaukee, USA), which was used to evaluate protein loading in each lane. After a 12 h incubation at 4°C, the membranes were washed with TBST and incubated with horseradish peroxidase-conjugated anti-rabbit secondary antibodies (Millipore; 1:5000 with TBST) for 1 h. Finally the images were captured by densitometry (Bio-Rad, Hercules, USA).

### Quantitative real-time polymerase chain reaction (qPCR)

*In vivo*, total RNA was extracted from hippocampus using Trizol reagent (Invitrogen) and cDNA was obtained by using a QuantiTect Reverse Transcription Kit (Qiagen, Hilden, Germany) according to the manufacturer’s instructions. The primer sequences were as follows: BDNF forward: TCCCTggCTgACACTTTTgA; BDNF reverse: CCAgCAgAAAgAgCAgAggA; β-actin forward: AgATCCTgACCgAgCgTggC; β-actin reverse: CCAgggAggAAgAggATgCg. qPCR of BDNF mRNA expression was carried out by using SYBR green mix (Newbioindustry, Beijing, China) and analyzed using the 2^−ΔΔCt^ method.

For ex-vivo experiments, after T cells from rats’ spleens were cultured for 48 h in media or stimulated with GA (50 μg/ml), T cell supernatants were collected and subjected to qPCR.

### Enzyme-linked immunosorbent assay (ELISA)

Each hippocampus was dissected from brain tissue on ice and then homogenized with iced PBS to a 10% (w/v) homogenate. The activities of IFN-γ, IL-10, IL-4, IL-6 and TNF-α were determined using the multi-analyte ELISArray Kit (SABiosciences, Frederick, MD, USA) according to the manufacturer’s instructions. Protein concentration was determined by the BCA (bicinchonininc acid) method.

### Evaluation and statistical analysis

The SPSS 17.0 analysis software was applied to compare differences among groups. The average latency time were analyzed with two-way repeated analysis of variance (ANOVA); The ex-vivo experiments of BDNF was evaluated by Student´s t-test (T); Other data sets were assessed by one-way ANOVA, and multiple comparisons between the groups were analyzed using the S-N-K method. Data are presented as means ± SEM. For all statistical analyses, significant differences were set at *P* < 0.05.

## Additional Information

**How to cite this article**: Chen, L. *et al*. T cell immunity to glatiramer acetate ameliorates cognitive deficits induced by chronic cerebral hypoperfusion by modulating the microenvironment. *Sci. Rep*. **5**, 14308; doi: 10.1038/srep14308 (2015).
